# 7-Acetylsinumaximol B Induces Apoptosis and Autophagy in Human Gastric Carcinoma Cells through Mitochondria Dysfunction and Activation of the PERK/eIF2α/ATF4/CHOP Signaling Pathway

**DOI:** 10.3390/md16040104

**Published:** 2018-03-26

**Authors:** Tsung-Chang Tsai, Kuei-Hung Lai, Jui-Hsin Su, Yu-Jen Wu, Jyh-Horng Sheu

**Affiliations:** 1 Department of Marine Biotechnology and Resources, National Sun Yat-sen University, Kaohsiung 80424, Taiwan; n833_d10@yahoo.com.tw; 2 Department of Nephrology, Antai Medical Care Corporation Antai Tian-Sheng Memorial Hospital, Pingtung 92842, Taiwan; 3 National Museum of Marine Biology & Aquarium, Pingtung 94450, Taiwan; mos19880822@gmail.com (K.-H.L.); x2219@nmmba.gov.tw (J.-H.S.); 4 Department of Biological Technology, Mei-ho University, Pingtung 91202, Taiwan; 5 Graduate Institute of Natural Products, Kaohsiung Medical University, Kaohsiung 80708, Taiwan; 6 Department of Medical Research, China Medical University Hospital, China Medical University, Taichung 40402, Taiwan; 7 Frontier Center for Ocean Science and Technology, National Sun Yat-sen University, Kaohsiung 80424, Taiwan; 8 Doctoral Degree Program in Marine Biotechnology, National Sun Yat-sen University, Kaohsiung 80424, Taiwan

**Keywords:** 7-acetylsinumaximol B, human gastric cancer cells, endoplasmic reticulum stress, mitochondrial inactivation, autophagy

## Abstract

The 7-Acetylsinumaximol B (7-AB), a bioactive cembranoid, was originally discovered from aquaculture soft coral *Sinularia sandensis*. The current study investigated the anti-proliferative property of 7-AB towards the NCI-N87 human gastric cancer cell line. An MTT cell proliferative assay was applied to evaluate cell survival, and immunofluorescence staining and western blotting were employed to analyze the effects of 7-AB on autophagy and apoptosis. Our results showed that 7-AB exerted a concentration-dependent anti-proliferative effect on NCI-N87 cells, and fluorescence staining indicated that the effect was due to the apoptosis induced by 7-AB. In addition, the 7-AB-induced anti-proliferation towards NCI-N87 cells was associated with the release of cytochrome *c* from mitochondria, activation of pro-apoptotic proteins (such as caspase-3/-9, Bax and Bad), and inhibition of anti-apoptotic proteins (Bcl-2, Bcl-xL, and Mcl-1). The 7-AB treatment also triggered endoplasmic reticulum (ER) stress, leading to activation of the PERK/elF2α/ATF4/CHOP apoptotic pathway. Furthermore, 7-AB initiated autophagy in NCI-N87 cells and induced the expression of autophagy-related proteins, including Atg3, Atg5, Atg7, Atg12, LC3-I, and LC3-II. Taken together, our findings suggested that 7-AB has the potential to be further developed as a useful anti-cancer or adjuvant agent for the treatment of human gastric cancer.

## 1. Introduction

Gastric cancer owns the fifth highest mortality rate of the top ten causes of cancer death, which equates to 15.76% of the total incidence of cancer in Taiwan. The causes of gastric cancer are still unclear, but associations with race, lifestyle, culture, diet, and environmental factors have been suggested. People with early-stage gastric cancer rarely experience symptoms. In the later stages, gastric cancer often does not cause specific symptoms, or the symptoms might be vague when they do occur. Abdominal pain is the main symptom, other symptoms being loss of appetite, weight loss, vomiting, and black stools [1]. Although the mortality rate of gastric cancer is declining, it remains a very serious illness and should not be neglected. Surgery, targeted therapy, radiation therapy, and chemotherapy are the major treatments for gastric cancer currently [2].

Chemotherapy involves the use of drugs that inhibit the proliferation of cancer or directly destroy cancer cells to achieve the purpose of cancer treatment. The process involves a mechanism that induces apoptosis pathways in cancer cells, causing programmed cell death. This is currently the most widely studied and most effective way of treating cancer [3]. Induction of apoptosis involves both intrinsic and extrinsic pathways. The intrinsic pathway is caused by intracellular events related to mitochondria and the endoplasmic reticulum (ER) [4,5]. Pro-apoptotic members of the Bcl-2 family, such as Bax, play important roles in apoptosis by altering the mitochondrial membrane potential and triggering the release of mitochondrial factors that activate apoptosis. Bax also induces the release of cytochrome *c* from mitochondria to the cytoplasm, subsequently activating caspase-9 and causing downstream activation of caspase 3, which results in cleavage of poly (ADP-ribose) polymerase-1 (PARP-1) [6,7].

In addition, a process called autophagy occurs in all eukaryotic cells to maintain normal cell development, as it ensures a well-controlled balance between anabolism and catabolism. When cells receive stimulation, autophagy occurs quickly, which helps cells to survive, while excessive autophagy may also cause cell death. Therefore, autophagy has been suggested to have dual roles in cells, acting as a mechanism of both promoting and preventing cell survival [8]. Autophagy initiates a process of non-apoptotic death that inhibits tumorigenesis, and therefore can slow down tumor progression [9].

Over the years, malignant neoplasms have remained in the top 10 leading causes of death in most countries. Currently, surgical resection and chemotherapy are still the most common methods used to treat cancer. However, they all have some limitations and adverse effects. Therefore, we aimed to develop new anti-cancer drugs that have highly specific cytotoxic effects on cancer cells and do not cause resistance in cancer cells.

Soft corals are rich in compounds with bioactivities, such as cytotoxicity and anti-inflammatory effects. A sphingosine derivative and a cembrenoid diterpene, lobohedleolide, were isolated from soft corals *Sinularia crassa* and *Lobophytum* species by Radhika et al. [10] in 2005, and were shown to possess anti-inflammatory activity in an animal model. The 7-Acetylsinumaximol B (7-AB), formerly isolated from aquaculture soft coral *Sinularia sandensis* [11], is a recently identified natural compound that exhibits basic-type cembrane skeleton [12] with two additional cyclization in its 14-membered ring. Cembrane-type compounds have been found to possess potent anti-inflammatory and anti-cancer activities. In 1996, cembranoid diterpenes were isolated by Duh and coworkers [13] from the soft coral *Sinularia gibberosa*; in vitro experiments showed its cytotoxic effects on mouse leukemia cells (P388), human lung adenocarcinoma cells (A549), human colorectal adenocarcinoma cells (HT-29), and human oral cancer cells (KB). In 2010, other new cembranoids, sarcocrassocolides, were purified from soft coral *Sarcophyton crassocaule* by Lin et al. [14]. These compounds were found to exhibit cytotoxicity towards a group of cancer cell lines, including human medulloblastoma cell lines (DAOY), human laryngeal carcinoma cells (Hep-2), human breast adenocarcinoma cells (MCF-7), and human colon carcinoma cells (WiDr). In addition, at low concentrations, they exhibit anti-inflammatory activity and inhibit the expression of iNOS pro-inflammatory protein. As 7-AB has not been studied in relation to gastric cancer, we therefore utilized in vitro human gastric carcinoma NCI-N87 cell model to assess the anti-proliferative effect of this compound, and evaluated the potential for its development as a new nature-derived agent for the treatment of gastric cancer.

## 2. Results

### 2.1. Anti-Proliferative Effect of 7-Acetylsinumaximol B (7-AB) on NCI-N87 Cells

**Figure 1:**
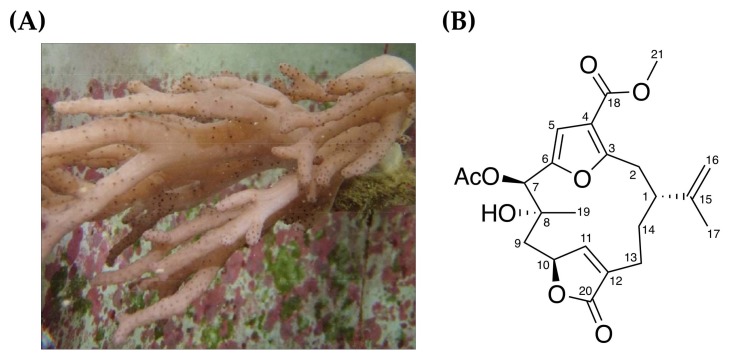
(**A**) The aquaculture soft coral *Sinularia sandensis* and (**B**) its bioactive component 7-Acetylsinumaximol B (7-AB).

**Figure 2:**
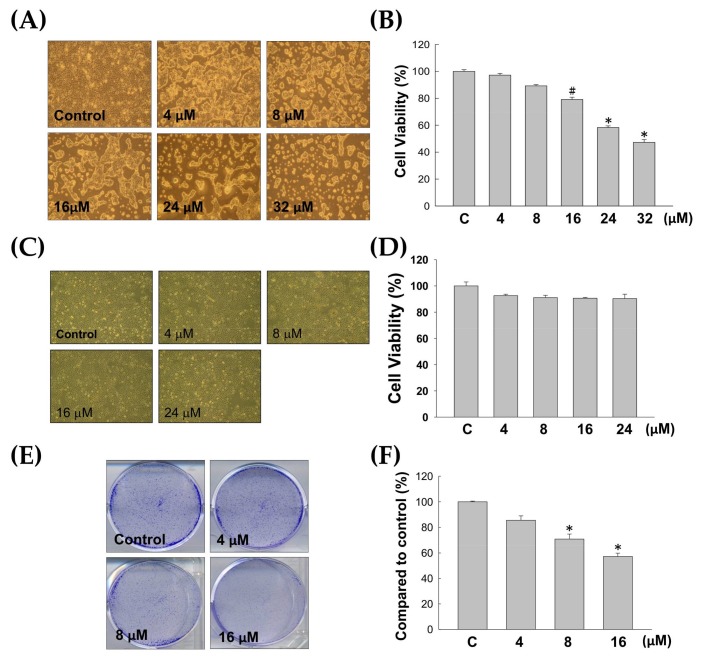
Evaluation of the anti-proliferative effects of 7-AB on NCI-N87 cells. (**A**) Morphological change of NCI-N87 cells upon 7-AB treatment. NCI-N87 cells were treated with DMSO as the control or 7-AB at final concentrations of 4, 8, 16, 24, and 32 μM, respectively, followed by observation of the morphology of the cells under inverted light microscopy; (**B**) The viability of NCI-N87 cells was concentration-dependently suppressed by treatment with 7-AB at final concentrations of 4, 8, 16, 24, and 32 μM for 24 h. (* *p* < 0.001, ^#^ *p* < 0.05) Inhibitory effects on cell proliferation were assessed by MTT assay, as described in Materials and Methods; (**C**) Morphology and (**D**) cell survival of HaCaT cell under the treatment of 7-AB; (**E**) In colony formation assay, NCI-N87 cells were clearly reduced (upon 16 μM of 7-AB treatment) as compared with control cells at 100× magnification; (**F**) 7-AB also showed concentration-dependent (4, 8, and 16 μM) inhibition on NCI-N87 cell colony formation. (* *p* < 0.001).

Cell morphology analysis, MTT cell viability assays and colony formation assays were performed in this study to investigate the anti-proliferative effect of 7-acetylsinumaximol B (7-AB) (Figure 1) on NCI-N87 cells. Cells were treated with 4, 8, 16, 24, and 32 μM of 7-AB for 24 h. As shown in Figure 2A, with an increasing 7-AB concentration, the cell morphology and growth changed substantially. To examine whether the changes were due to the anti-proliferative effect of 7-AB on the cells, we used MTT assays to examine the cell viability after 24 h of 7-AB treatment. Our results showed that cell viability decreased as the concentration of 7-AB increased (Figure 2B), indicating the anti-proliferative effect of 7-AB with the IC_50_ value of 30.28 μM. We then performed colony formation assays with cells treated with three different concentrations of 7-AB. At 48 h after the treatment, the percentages of cell colonies inhibition following treatment with 4, 8, and 16 μM of 7-AB were 86%, 71%, and 54%, respectively, as compared with cells treated with vehicle control (Figure 2E,F). Moreover, the effect of 7-AB on the human immortalized keratinocytes HaCaT cell was also carried out in order to determine its selectivity during the treatment. The exposure of HaCaT cell with increasing concentration (up to 24 μM) of 7-AB did not fairly affect its survival (Figure 2C,D). These results indicated that 7-AB possessed a significant anti-proliferative effect on NCI-N87 cells without considerable cytotoxicity toward normal cell.

### 2.2. 7-AB Induced Apoptosis in NCI-N87 Cells

**Figure 3:**
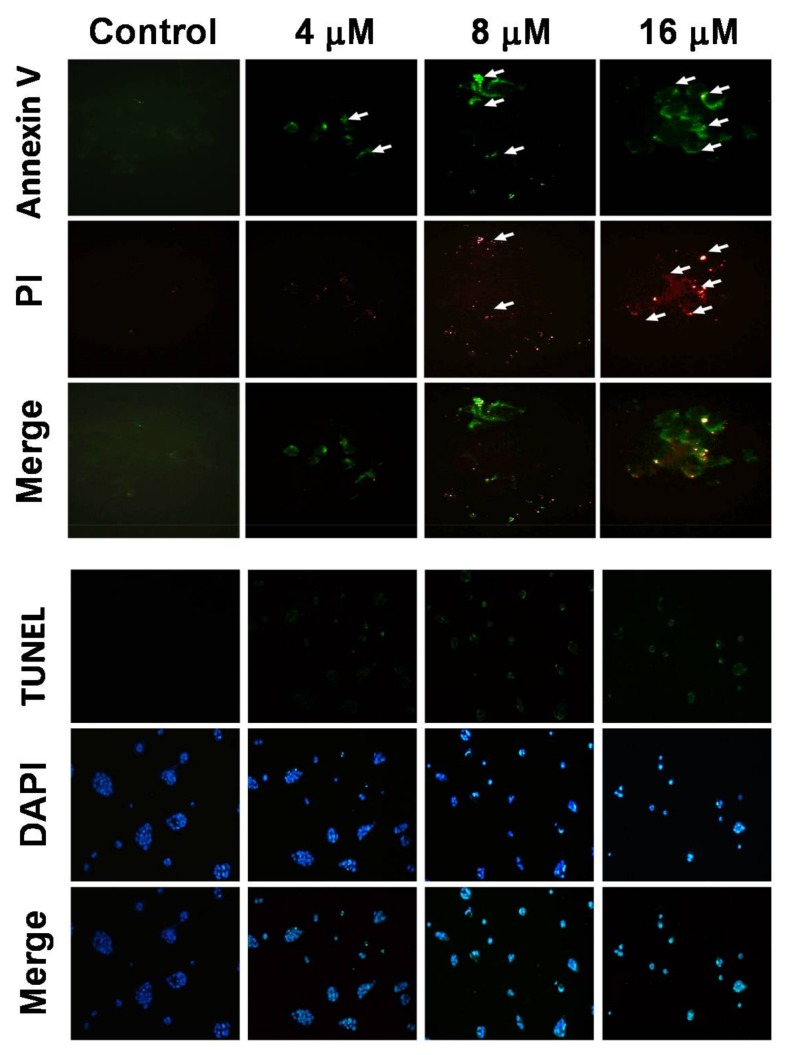
Appearance of apoptosis characteristics in 7-AB-treated NCI-N87 cells. Detection of externalization of phosphatidylserine (PS) from the cell membrane after 7-AB treatment by Annexin V-fluoresceinisothiocyanate (FITC)/propidium iodide (PI) staining. Annexin V-FITC and PI analysis of apoptotic NCI-N87 cells upon 7-AB treatment. NCI-N87 cells were stained with PI (red) and Annexin-V (green) after 7-AB treatment at final concentrations of 4, 8, and 16 μM. Detection of apoptotic cells by TUNEL and DAPI (blue) staining assay. NCI-N87 cells were treated with DMSO or 7-AB at final concentrations of 4, 8, and 16 for 24 h. Cells were harvested for TUNEL staining as described in the Materials and Methods section. NCI-N87 cells were treated with DMSO or 7-AB at final concentrations of 4, 8, and 16 μM for 24 h. After harvesting, cells were then fixed with paraformaldehyde for immunofluorescent staining. NCI-N87 cells were then stained with DAPI and visualized by fluorescent microscope.

NCI-N87 cells treated with 4, 8, and 16 μM of 7-AB were analyzed in terms of the level of apoptosis using Annexin V/propidium iodide (PI) assays. With Annexin V (green) and PI (red) double staining, the results showed that 7-AB increased both the Annexin V and PI levels as compared with cells treated with control, in which no staining was seen (Figure 3). In addition, TUNEL assay demonstrated that cells treated with increased 7-AB concentrations exhibited higher levels of apoptosis. DAPI staining also showed that following treatment with 8 μM of 7-AB, the nuclei of the cells began to shrink, and cells treated with 16 μM of 7-AB exhibited the presence of apoptotic bodies in the cells.

### 2.3. 7-AB Activates the Caspase Pathway to Induce Apoptosis

**Figure 4:**
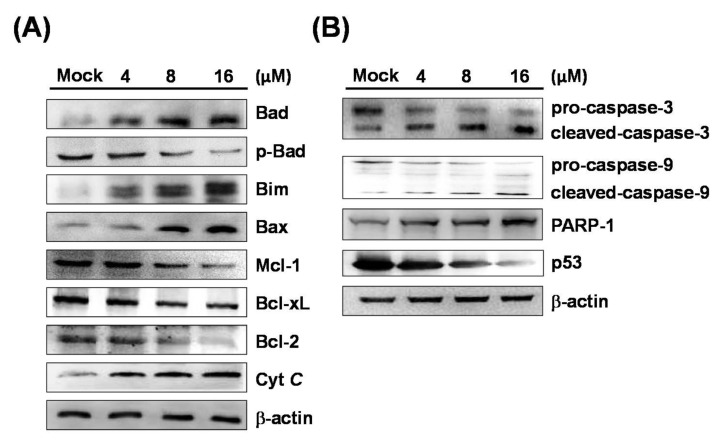
Effect of 7-AB on apoptosis induction via mitochondrial-associated pathway. NCI-N87 cells were treated with indicated concentrations (4, 8, and 16 μM) of 7-AB for 24 h, respectively. The expression on mitochondrial-associated apoptotic proteins was detected employing Western blotting analysis with indicated antibodies. The results demonstrated changes in (**A**) cytochrome *c*, Bax, Bcl-2, Bcl-xL, Mcl-1, Bim, Bad, and *p*-Bad; and (**B**) caspase-3, caspase-9, cleaved-caspase-3, cleaved-caspase-9, and PARP-1 expressions in NCI-N87 cells treated with 7-AB. β-actin was used as the internal protein loading control.

The process of apoptosis may involve intrinsic and extrinsic pathways. The intrinsic pathway is caused by stress from intracellular organelles, such as mitochondria and the endoplasmic reticulum (ER). Mitochondria are thought to be the regulatory center of apoptosis, and the relevant proteins that are involved in apoptotic pathways include caspase-3, caspase-9, PARP-1, cytochrome *c*, Bax, Bim, Bcl-xL, Mcl-1, and Bcl-2, Bad, and *p*-Bad. In order to study the mechanism of 7-AB-induced apoptosis in NCI-N87 cells, we performed Western blot analysis to explore the changes in these proteins after 7-AB treatment. As shown in Figure 4, cells treated with higher concentrations of 7-AB exhibited increased expressions of Bad, Bim, Bax, and cytochrome *c* protein, while the expression levels of *p*-Bad, Mcl-1, Bcl-xL, and Bcl-2 proteins were reduced with an increasing 7-AB concentration. The results demonstrated that 7-AB-induced apoptosis in NCI-N87 cells is associated with the expressions of Bcl-2 family proteins. In addition, our results also indicated increased Bax and cytochrome *c* expressions in the cells after 7-AB treatment, which suggested that 7-AB-induced apoptosis is mediated by inactivation of mitochondria. Thus, the finding implied that the balance between the Bax (pro-apoptotic member) and Bcl-2 (anti-apoptotic member) protein families plays a role in the regulation of cytochrome *c* release from mitochondria to the cytoplasm, and that the process may be key to further downstream caspase activities.

Caspases are a group of kinases that regulate cell survival and cell apoptosis. Once inactivation of mitochondria occurs, cytochrome *c* is released into the cytoplasm and activates caspase-3 and caspase-9, which consequently causes apoptosis. In order to investigate the relationship between apoptosis and caspase activation induced by 7-AB, we next studied caspases expressions, especially caspase-3 and caspase-9, in NCI-N87 cells treated with 7-AB using Western blotting. As shown in Figure 4, 7-AB treatment reduced the levels of pro-caspase-3 and pro-caspase-9, and boosted the amounts of cleaved caspase-3 and caspase-9. In addition, the activated PARP-1 protein level gradually increased after 7-AB treatment. The results suggested that 7-AB caused mitochondrial inactivation and increased caspase levels, leading to apoptosis in the cells.

### 2.4. 7-AB-Induced Apoptosis Is Mediated by an Increased Endoplasmic Reticulum Stress Response

**Figure 5:**
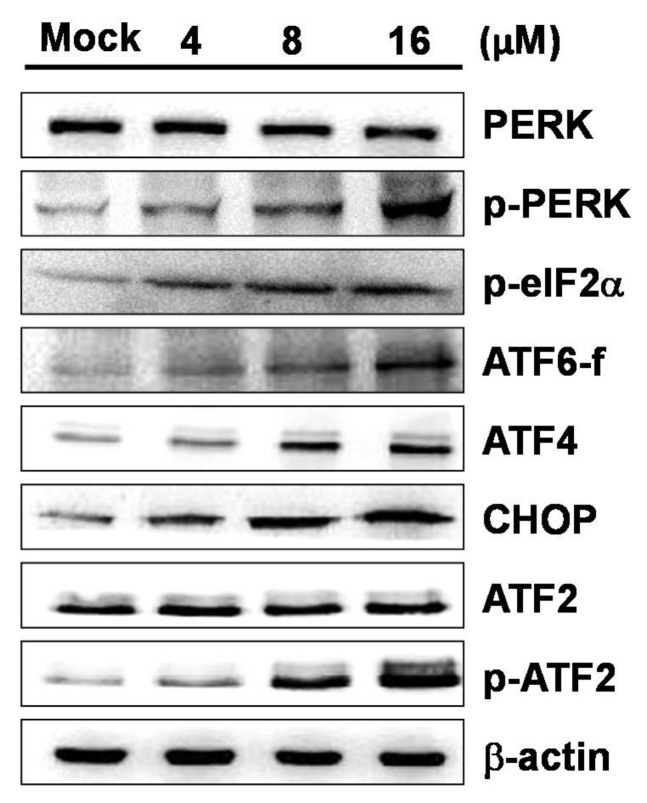
Effect of 7-AB on endoplasmic reticulum (ER) stress-associated apoptosis pathway. NCI-N87 cells were treated with indicated concentrations (4, 8, and 16 μM) of 7-AB for 24 h, respectively. Detection of ER stress-associated apoptosis proteins was carried out by Western blotting analysis using indicated antibodies. The results displayed expression changes in PERK, *p*-PERK, *p*-eIF2α, ATF4, ATF6, CHOP, ATF2, and *p*-ATF2 expressions in NCI-N87 cells treated with 7-AB. β-actin was used as the internal protein loading control.

The results of the experiments described above confirmed that mitochondrial inactivation caused by 7-AB plays a role in apoptosis, leading to increases in apoptosis-related proteins such as Bax, Bad, and cytochrome *c*. We next investigated the relationship between 7-AB-induced apoptosis and ER stress in NCI-N87 cells. When cells are under oxidative stress, accumulation of unfolded and misfolded proteins in the ER lumen causes ER stress. If the ER stress persists, it may activate downstream proteins, subsequently causing apoptosis through pathways mediated by PKR-like ER-associated kinase (PERK), inositol options enzyme-1 α (IRE1α), and activating transcription factor 6 (ATF6). By measuring the expressions of proteins associated with these pathways, we found that the expressions of phosphorylated PERK (*p*-PERK), phosphorylated eIF2α (*p*-eIF2α), ATF4, and CHOP were increased; the expression of ATF6, a key player in apoptosis, was also increased (Figure 5). The results suggested that the mechanism of 7-AB-induced apoptosis is mediated by the PERK/eIF2α/ATF4/CHOP stress response pathway in the ER.

### 2.5. 7-AB Causes Autophagy in NCI-N87 Cells

**Figure 6:**
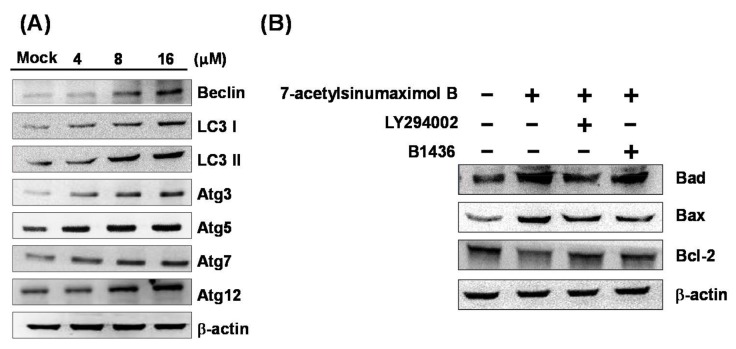
Effect of 7-AB on inducing apoptosis through the autophagy-associated apoptosis pathway. NCI-N87 cells were treated with indicated concentrations (4, 8, and 16 μM) of 7-AB for 24 h, respectively. (**A**) Investigation of autophagy-associated apoptosis proteins was executed by Western blotting analysis using indicated specific antibodies. The results revealed changes in the expressions of Beclin, LC3I, LC3II, Atg3, Atg5, Atg7, and Atg12 in NCI-N87 cells treated with 7-AB; (**B**) Moreover, the effect of LY294002 (20 μM) and B1436 (20 μM) pretreatments (treated with 8 μM 7-AB for 24 h) on anti-apoptotic and pro-apoptotic proteins were also evaluated. β-actin was used as the internal protein loading control.

In this study, we used Western blotting to analyze the expressions of autophagy-associated proteins, and aimed to identify whether 7-AB induced autophagy in the NCI-N87 cells. Our results demonstrated that with an increased 7-AB concentration, the expressions of autophagy-associated proteins, including Beclin-1, LC3-I, LC3-II, Atg3, Atg5, Atg7, and Atg12, were also augmented in NCI-N87 cells (Figure 6A). Moreover, in order to further determine the interplay of autophagy and apoptosis in the 7-AB-treated NCI-N87 cell, the apoptotic inhibitor (B1436) and autophagic (LY294002) inhibitor were employed (Figure 6B). The results revealed that both inhibitors abrogated the induction of the pro-apoptotic protein Bax and Bad as well as the suppression of anti-apoptotic protein Bcl-2 expression, indicating the pro-apoptotic effect of 7-AB-induced autophagy. These findings suggested that 7-AB treatment resulted in damage to organelles, which then generated cellular stress to induce autophagy and apoptosis in the cells.

## 3. Discussion

Apoptosis caused by mitochondrial inactivation is closely related to the Bcl-2 family proteins, in which the balance between the proapoptotic protein Bax and the anti-apoptotic protein Bcl-2, the gradual release of cytochrome *c* from mitochondria to the cytoplasm, and downstream caspase activation all play important roles [15,16]. In NCI-N87 cells treated with 7-AB, increased levels of Bax and cytochrome *c* indicated that 7-AB-induced apoptosis is associated with mitochondrial inactivation.

Caspases are a group of kinases that function as the primary drivers of apoptotic cell death. Previous studies have shown that when the initiation of mitochondrial inactivation leads to cytochrome *c* release into the cytoplasm, further activation of caspase-3 and caspase-9 occurs to promote apoptosis [17]. However, anti-apoptotic Bcl-2 family proteins in cells, such as Bcl-2, Bcl-xL, and Mcl-1, may prevent the intrinsic apoptosis signaling pathway and rupture the outer mitochondrial membrane, which indirectly or directly inhibits the activation of Bad and Bax [18]. Shimizu et al. [19] found that several caspases need to be cleaved to be active, and initiator caspases, such as caspase-8 or caspase-9, need to be active first in order to activate the executioner caspase, caspase-3, to induce apoptosis. Our results showed that the mitochondrial membrane potential decreased in NCI-N87 cells treated with 7-AB for 48 h, and Western blot analysis demonstrated that the expression of Bax was gradually increased, and the expression of Bcl-2/Bcl-xL was inhibited. In the meantime, Bax was translocated to the outer membrane of mitochondria, causing the release of cytochrome *c*, and further resulting in the activation of caspase-3 and caspase-9. Based on the findings from the aforementioned experiments, 7-AB causes mitochondrial inactivation that regulates the apoptosis pathway, which is key to its anti-proliferative effect. The ER is a multifunctional organelle, the primary functions of which are protein synthesis and folding; it also serves as an important location for Ca^2+^ storage and signaling for cells. Toth et al. [20] found that perturbations of the functions of the endoplasmic reticulum result in ER stress, which can activate the expression of ER stress transducers, PERK and ATF-6, to induce transcription of CHOP, which upregulates the PERK/ATF4/CHOP pathway and promotes cell apoptosis. The apoptotic effect accentuated by CHOP is closely associated with the decrease in Bcl-2 expression [21], whereas expression of ATF-4 protein is positively correlated with that of CHOP. Therefore, when ATF4 induces downstream CHOP protein expression, the cells will gradually switch from autophagy to apoptosis [22]. At the same time, when PERK separates from its ER chaperone molecule, it causes PERK oligomerization in the ER lumen, resulting in its phosphorylation and promoting downstream eIF2α phosphorylation [23]. Phosphorylated eIF2α and CHOP are important indicators of the ER stress adaptive response and apoptotic process, respectively. During the early stages of the ER stress response, in order to protect normal cell function, the main response is to prevent unfolded protein synthesis or to promote correct protein folding. When the adaptive response is not able overcome the stress, the apoptotic pathway will be promoted in cells [24]. Tasdemir and colleagues [25] showed that when the expression of p53 protein decreases, autophagy increases in the cells, and cytoplasmic p53 may downregulate autophagy in cells. In our current study, a similar phenomenon was observed.

Autophagy is an important mechanism that cells use to disassemble unnecessary or dysfunctional organelles or proteins by sending them to the lysosome, where they are degraded and recycled. Cells have evolved several mechanisms to deal with life-threatening stress, such as starvation. Among these mechanisms, autophagy is known to be an immune defense mechanism, and also plays a key role in the response of cells to starvation. In addition to the destruction of cellular organelles, the mechanism may induce signals to cause apoptosis if the stress is too severe. Cells are destined to die via apoptosis, while autophagy may control cell survival or death, depending on the accumulation of aggregate proteins in the damaged cells. Therefore, although apoptosis and autophagy are different processes, each serving a distinct purpose, their regulation is closely connected, and a fine balance between them controls the fate of cells. Autophagy stimulated by ER stress occurs quickly to help the cells survive, while imbalance or over-activation of autophagy causes cell death. Thus, autophagy is known to have a dual role, as it controls both cell survival and death [8].

Luo et al. [26] found that the JNK signaling pathway can phosphorylate Bcl-2 family proteins and affect the binding of Beclin-1 to regulate autophagy, and the process is mediated though its ability to phosphorylate Bcl-2 family proteins. The effect of JNK signaling on autophagy may be mediated through two pathways: (1) JNK-1 can induce Bcl-2/Bcl-xL phosphorylation, which then results in Beclin-1 separating from the Bcl-2/Bcl-xL complex; (2) Bcl-2/Bcl-xL phosphorylation may influence the interaction of Beclin-1 with the BH3 domain. Zhou et al. [27] showed that Bcl-2 phosphorylation may induce Beclin-1 to separate from its complex, leading to the development of autophagy. Another study found that reduction of the Atg7 phagocytic protein downregulates autophagy. In addition, inhibition of JNK expression reduces Atg7 expression, suggesting that the JNK pathway regulates autophagy via controlling Atg7 expression [28]. Radoshevich et al. [29] demonstrated that Atg7 protein also acts as an enzyme that can activate Atg12 protein, which results in the glycine on the activated Atg12 forming a covalent link with the lysine on Atg3, leading to formation of an Atg12–Atg3 conjugate. The conjugate restricts mitochondrial mass and promotes mitochondrial fusion, which may disrupt the intracellular homeostatic balance and lead to cell death. In eukaryotes, autophagy is an intracellular degradation process in which a large number of intracellular components are degraded. It starts with the formation of a double-membrane vesicle called an autophagosome, which encapsulates cytoplasmic components into a spherical structure. In 2007, Hanada et al. [30] found that autophagosome formation requires Atg12–Atg5 conjugation for expansion of the autophagosomal membrane, and the conjugate interacts with both Atg3 and phosphatidylethanolamine-containing liposomes to promote protein-lipid conjugation in autophagy. In addition to the Atg12 system, microtubule-associated protein 1 light chain 3 (LC3) and it relevant proteins are also involved in the formation of autophagosomes. LC3 undergoes post-translational modifications to form two isoforms: LC3-I and LC3-II. LC3-I is formed from newly-synthesized LC3 by removing 22 amino acids from the C-terminal, and LC3-II is generated from the conversion of a small portion of LC3-I. LC3-I is distributed in the cytoplasm, while LC3-II is present both inside and outside the autophagosomal membranes. Moreover, the level of LC3-II in the cells is correlated with the extent of autophagosome formation. As LC3-II was the first protein identified in mammalian cells to be associated with the autophagosomal membrane, it is therefore considered to be a marker of autophagy in mammals [31,32]. Furthermore, p53 has been shown to inhibit autophagy in starvation-induced autophagy, and thus inhibition of p53 represents a potential anti-cancer mechanism of new drugs being developed for cancer treatment. Also, glucose starvation induces p53 phosphorylation, which can subsequently regulate the activation of autophagy to support cell survival [33,34].

The cross-talk mechanisms between autophagic and apoptotic pathways, including inducer and signaling pathways, has been demonstrated in the previous reports [35,36]. Also, the autophagy required protein, Atg5, was found to cause the cell death by operating the apoptotic pathway without activating autophagic pathways [37,38]. Furthermore, Bcl-2 and Bcl-xL, the well-distinguished anti-apoptotic proteins, appeared to be important modulators of autophagy via binding and inhibiting Beclin 1. In the current study, the expression of Bcl-2, Bcl-xL, and Mcl-1 were found to be suppressed under the treatment of 7-AB in NCI-N87 cancer cell, while the levels of Atg5, Beclin 1, Bax, Bad, Bim were promoted, which demonstrated that 7-AB may induce apoptosis and autophagy through the dysfunction of mitochondria. It has been reported that the eIF2/ATF4 pathway is essential for ER stress-induced autophagy gene such as Beclin 1 and Atg12 expression [39]. In this study, we demonstrated 7-AB induced ER stress-autophagy cross-talk comes from activation PERK/eIF2/ATF4/CHOP pathway on NCI-N87 gastric cancer cells.

## 4. Materials and Methods

### 4.1. Chemicals and Reagents

The 7-Acetylsinumaximol B (7-AB) was obtained from J.S. Su’s lab using the isolating strategy reported in the previous study [11]. MTT, LY294002 (autophagy inhibitor), B1436 (Bax-mediated apoptosis inhibitor), and goat anti-rabbit β-Actin antibody were purchased from Sigma-Aldrich (St. Louis, MO, USA). Antibodies were obtained from ProteinTech Group (Chicago, IL, USA), Assay BioTech (San Francisco, CA, USA), and Epitomics (Burlingame, CA, USA).

### 4.2. Cell Culture

Human gastric carcinoma NCI-N87 cells and human immortalized keratinocytes HaCaT cells were cultured in DMEM containing 4 mM l-glutamine, 1.5 g/L sodium bicarbonate, 4.5 g/L glucose, 100 U/mL penicillin, 100 μg/mL streptomycin, 1mM sodium pyruvate, and 10% FBS (*v*/*v*). The cells were grown at 37 °C in a humidified incubator in an atmosphere of 5% CO_2_. Active compound 7-acetylsinumaximol B (7-AB) was purified from cultured-type soft coral *Sinularia sandensis*. The cells were treated with different concentrations of 7-AB (4, 8, 16, 24, and 32 μM) or with DMSO vehicle. After incubation for a suitable duration for each type of experiment, the cells were analyzed. All the experiments were repeated at least three times.

### 4.3. MTT Cell Survival Assay

MTT (3-[4,5-Dimethylthiazol-2-yl]-2,5-diphenyltetrazolium bromide) is a yellow tetrazolium salt that may enter cells, and succinic dehydrogenase enzyme in live cells oxidizes MTT to yield a water-insoluble purple formazan crystal. This study used MTT assays to examine the cell survival and proliferation of NCI-N87 and HaCaT cells after 7-AB treatment. Briefly, cells at 1 × 10^5^ cells/mL were seeded onto 96-well plates (150 μL/well) and incubated with different concentrations of 7-AB (4, 8, 16, 24, and 32 μM) for 24 h. After adding 50 μL MTT solution (1 mg/mL in PBS) to each well, the culture was incubated at 37 °C for 4 h, following which 200 μL DMSO were added to dissolve the formazan. The plate was read on an ELISA microplate reader at an absorbance of 595 nm.

### 4.4. Colony Formation Assay

NCI-N87 cells at 2000 cells/well were seeded in 24-well plates. After the cells had been incubated for 24 h, the cell culture medium was then changed to fresh medium that contained 7-AB (4, 8, 16, 24, and 32 μM), and incubated for a further 10 days. After culture for 10 days, media were removed and colonies were washed with PBS. The colonies were fixed with methanol for 15 min and stained with 0.15% crystal violet for 10 min. The colonies were counted and scanned with a high-resolution scanner Scan Maker 9800XL (MiCROTEK, Hsinchu, Taiwan).

### 4.5. Annexin V/Propidium Iodide (PI) Staining

The Annexin V-FITC Apoptosis Detection Kit (Strong Biotech Corporation, Taipei, Taiwan) was employed for Annexin V/propidium iodide (PI) staining experiment based on the provided protocols. For the detailed process, cells were seeded in a six-well plate at 3 × 10^5^ cells/well. After adhesion, the NCI-N87 cells were treated with 7-AB for 12 h and harvested by trypsinization. Cells were centrifuged for 5 min at 2000 rpm, then the cell pellet was resuspended in 1 mL of PBS. The cells were washed with PBS three times and then mixed with 100 µL Annexin V binding buffer, continued with the addition of 5 µL each of Annexin V and PI. After incubation for 10–15 min in the darkness, Annexin V binding buffer was added to bring the total volume to 1 mL.

### 4.6. TUNEL/DAPI Staining

TUNEL/DAPI staining was applied with the In Situ Cell Death Detection Kit, Fluorescein (Roche Diagnostics, Indianapolis, IN, USA), and DAPI (Sigma-Aldrich, St. Louis, MO, USA). The NCI-N87 gastric cancer cell lines were cultured on coverslips at 1 × 10^4^ cells/well. After adhesion, the cells were treated with 7-AB for 24 h. After removal of cell culture media, the cells were fixed with 4% paraformaldehyde for 20 min at room temperature (rt). The cells were then washed with PBS for 3 times, and the cell membranes were permeated with 0.1% Triton-X 100 on ice for 2 min. Afterwards, the cells were washed with cold PBS three times. Thirty microliters of precooled ice-cold TUNEL staining solution and enzyme mixed at a 1:19 ratio were added to the cells, followed by incubation for 1 h at 37 °C. The cells were washed with PBS three times, and the cells were stained with 1 µL of DAPI in 1 mL of PBS for 10 s. The samples were mounted onto slides and examined under a fluorescent microscope.

### 4.7. Protein Extraction and Estimation

Cells were treated with different concentrations of 7-AB (4, 8, and 16 μM) or control for 24 h, and then lysed to extract proteins using Cell Extraction Buffer (BioSource International, Camarillo, CA, USA) containing a protease inhibitor cocktail (Sigma-Aldrich, St. Louis, MO, USA). The cell lysate was centrifuged at 12,000× *g* for 10 min, and the supernatant was collected and mixed with 3 times the volume of ice cold 10% TCA/acetone to precipitate proteins overnight. The solution was then centrifuged at 8000× *g*, and the pellet was collected and dissolved in protein buffer (containing 6 M urea, 2 M thiourea, 0.5% CHAPS, 0.5% IPG, 20 mM DTT, and 0.002% bromophenol blue). The protein contents of the dissolved protein samples were then quantified.

### 4.8. Western Blot Analysis

For Western blot analysis, 20 μg of total protein of each sample were separated by SDS-PAGE, and transferred onto PVDF membrane at 400 mA for 1.5 h. After transfer, the membrane was blocked with blocking solution, then incubated with the primary antibody at 4 °C overnight. The blot was then washed with PBST (10 mM NaH_2_PO_4_, 130 mM NaCl, 0.05% Tween 20) three times, and incubated with horseradish peroxidase-conjugated secondary antibody (1:5000) for 1 h at room temperature. After washing three times in PBST, the blot was developed using Pierce ECL Western Blotting Reagents (Thermo Fisher Scientific, Waltham, MA, USA).

### 4.9. Statistical Analysis

Data obtained from the aforementioned experiments were analyzed by analysis of variance (ANOVA) and Tukey’s test using SPSS14.0 software (IBM, Endicott, NY, USA). A *p* value < 0.05 was considered to indicate a statistically-significant difference.

## 5. Conclusions

In the present study, 7-acetylsinumaximol B (7-AB) isolated from aquaculture soft coral *Sinularia sandensis* was identified to be a cembrane-type active compound. We tested the effects of 7-AB on human gastric cancer NCI-N87 cells and found that 7-AB induced apoptosis and autophagy in NCI-N87 cells. In addition to mitochondrial inactivation that occurred in the apoptosis pathway, the PERK/eIF2α/ATF4/CHOP signaling pathway was induced by ER stress. This was the first study to explore the anti-cancer activity of 7-AB. Our results clearly showed that 7-AB has an anti-proliferative effect on human gastric cancer cells, and the mechanism is associated with autophagy-related cell death. Furthermore, mitochondrial inactivation and ER stress also play key roles in 7-AB-induced apoptosis. Our findings indicated that active compound 7-AB derived from soft coral may be a good candidate for new drug development for the treatment of gastric cancer.

## References

[1] Ko F. (2006). Overview of gastric cancer. Taipei City Med. J..

[2] Orditura M., Galizia G., Sforza V., Gambardella V., Fabozzi A., Laterza M.M., Andreozzi F., Ventriglia J., Savastano B., Mabilia A. (2014). Treatment of gastric cancer. World J. Gastroenterol..

[3] Liu C.I., Wang R.Y., Lin J.J., Su J.H., Chiu C.C., Chen J.C., Chen J.Y., Wu Y.J. (2012). Proteomic profiling of the 11-dehydrosinulariolide-treated oral carcinoma cells Ca9-22: Effects on the cell apoptosis through mitochondrial-related and er stress pathway. J. Proteome.

[4] Denicourt C., Dowdy S.F. (2004). Medicine. Targeting apoptotic pathways in cancer cells. Science.

[5] Liao C.T., Chang J.T., Wang H.M., Ng S.H., Hsueh C., Lee L.Y., Lin C.H., Chen I.H., Huang S.F., Cheng A.J. (2008). Analysis of risk factors of predictive local tumor control in oral cavity cancer. Ann. Surg. Oncol..

[6] Nicholson D.W., Thornberry N.A. (2003). Apoptosis. Life and death decisions. Science.

[7] Amarante-Mendes G.P., Naekyung Kim C., Liu L., Huang Y., Perkins C.L., Green D.R., Bhalla K. (1998). Bcr-Abl exerts its antiapoptotic effect against diverse apoptotic stimuli through blockage of mitochondrial release of cytochrome C and activation of caspase-3. Blood.

[8] Rabinowitz J.D., White E. (2010). Autophagy and metabolism. Science.

[9] Edinger A.L., Thompson C.B. (2004). Death by design: Apoptosis, necrosis and autophagy. Curr. Opin. Cell Biol..

[10] Radhika P., Rao P.R., Archana J., Rao N.K. (2005). Anti-inflammatory activity of a new sphingosine derivative and cembrenoid diterpene (lobohedleolide) isolated from marine soft corals of *Sinularia crassa* tixier-durivault and *Lobophytum* species of the andaman and nicobar islands. Biol. Pharm. Bull..

[11] Tsai T.C., Chen H.Y., Sheu J.H., Chiang M.Y., Wen Z.H., Dai C.F., Su J.H. (2015). Structural elucidation and structure-anti-inflammatory activity relationships of cembranoids from cultured soft corals *Sinularia sandensis* and *Sinularia flexibilis*. J. Agric. Food Chem..

[12] Lai K.H., You W.J., Lin C.C., El-Shazly M., Liao Z.J., Su J.H. (2017). Anti-inflammatory cembranoids from the soft coral *Lobophytum crassum*. Mar. Drugs.

[13] Duh C., Hou R. (1996). Cytotoxic cembranoids from the soft corals *Sinularia gibberosa* and *Sarcophyton trocheliophorum*. J. Nat. Prod..

[14] Lin W.Y., Su J.H., Lu Y., Wen Z.H., Dai C.F., Kuo Y.H., Sheu J.H. (2010). Cytotoxic and anti-inflammatory cembranoids from the dongsha atoll soft coral *Sarcophyton crassocaule*. Bioorg. Med. Chem..

[15] Shankar S., Srivastava R.K. (2007). Bax and bak genes are essential for maximum apoptotic response by curcumin, a polyphenolic compound and cancer chemopreventive agent derived from turmeric, curcuma longa. Carcinogenesis.

[16] Wang H.L., Yeh T.H., Chou A.H., Kuo Y.L., Luo L.J., He C.Y., Huang P.C., Li A.H. (2006). Polyglutamine-expanded ataxin-7 activates mitochondrial apoptotic pathway of cerebellar neurons by upregulating Bax and downregulating Bcl-x(L). Cell Signal..

[17] Chen Y.J., Su J.H., Tsao C.Y., Hung C.T., Chao H.H., Lin J.J., Liao M.H., Yang Z.Y., Huang H.H., Tsai F.J. (2013). Sinulariolide induced hepatocellular carcinoma apoptosis through activation of mitochondrial-related apoptotic and perk/eif2alpha/atf4/chop pathway. Molecules.

[18] Adams J.M., Cory S. (2007). Bcl-2-regulated apoptosis: Mechanism and therapeutic potential. Curr. Opin. Immunol..

[19] Shimizu H., Banno Y., Sumi N., Naganawa T., Kitajima Y., Nozawa Y. (1999). Activation of p38 mitogen-activated protein kinase and caspases in uvb-induced apoptosis of human keratinocyte hacat cells. J. Investig. Dermatol..

[20] Toth A., Nickson P., Mandl A., Bannister M.L., Toth K., Erhardt P. (2007). Endoplasmic reticulum stress as a novel therapeutic target in heart diseases. Cardiovasc. Hematol. Disord. Drug Targets.

[21] Doyle K.M., Kennedy D., Gorman A.M., Gupta S., Healy S.J., Samali A. (2011). Unfolded proteins and endoplasmic reticulum stress in neurodegenerative disorders. J. Cell. Mol. Med..

[22] Matsumoto H., Miyazaki S., Matsuyama S., Takeda M., Kawano M., Nakagawa H., Nishimura K., Matsuo S. (2013). Selection of autophagy or apoptosis in cells exposed to er-stress depends on atf4 expression pattern with or without chop expression. Biol. Open.

[23] Gregor M.F., Hotamisligil G.S. (2007). Thematic review series: Adipocyte biology. Adipocyte stress: The endoplasmic reticulum and metabolic disease. J. Lipid Res..

[24] Xu C., Bailly-Maitre B., Reed J.C. (2005). Endoplasmic reticulum stress: Cell life and death decisions. J. Clin. Investig..

[25] Tasdemir E., Maiuri M.C., Galluzzi L., Vitale I., Djavaheri-Mergny M., D’Amelio M., Criollo A., Morselli E., Zhu C., Harper F. (2008). Regulation of autophagy by cytoplasmic p53. Nat. Cell Biol..

[26] Luo S., Rubinsztein D.C. (2013). Bcl2l11/bim: A novel molecular link between autophagy and apoptosis. Autophagy.

[27] Zhou F., Yang Y., Xing D. (2011). Bcl-2 and bcl-xl play important roles in the crosstalk between autophagy and apoptosis. FEBS. J..

[28] Wong C.H., Iskandar K.B., Yadav S.K., Hirpara J.L., Loh T., Pervaiz S. (2010). Simultaneous induction of non-canonical autophagy and apoptosis in cancer cells by ros-dependent erk and jnk activation. PLoS ONE.

[29] Radoshevich L., Murrow L., Chen N., Fernandez E., Roy S., Fung C., Debnath J. (2010). Atg12 conjugation to atg3 regulates mitochondrial homeostasis and cell death. Cell.

[30] Hanada T., Noda N.N., Satomi Y., Ichimura Y., Fujioka Y., Takao T., Inagaki F., Ohsumi Y. (2007). The atg12-atg5 conjugate has a novel e3-like activity for protein lipidation in autophagy. J. Biol. Chem..

[31] Kabeya Y., Mizushima N., Ueno T., Yamamoto A., Kirisako T., Noda T., Kominami E., Ohsumi Y., Yoshimori T. (2000). Lc3, a mammalian homologue of yeast apg8p, is localized in autophagosome membranes after processing. EMBO. J..

[32] Tanida I., Ueno T., Kominami E. (2004). Lc3 conjugation system in mammalian autophagy. Int. J. Biochem. Cell Biol..

[33] Feng Z., Zhang H., Levine A.J., Jin S. (2005). The coordinate regulation of the p53 and mtor pathways in cells. Proc. Natl. Acad. Sci. USA.

[34] Scherz-Shouval R., Weidberg H., Gonen C., Wilder S., Elazar Z., Oren M. (2010). P53-dependent regulation of autophagy protein LC3 supports cancer cell survival under prolonged starvation. Proc. Natl. Acad. Sci. USA.

[35] Levine B., Yuan J. (2005). Autophagy in cell death: An innocent convict?. J. Clin. Investig..

[36] Rubinstein A.D., Kimchi A. (2012). Life in the balance—A mechanistic view of the crosstalk between autophagy and apoptosis. J. Cell Sci..

[37] Pyo J.O., Jang M.H., Kwon Y.K., Lee H.J., Jun J.I., Woo H.N., Cho D.H., Choi B., Lee H., Kim J.H. (2005). Essential roles of atg5 and fadd in autophagic cell death: Dissection of autophagic cell death into vacuole formation and cell death. J. Biol. Chem..

[38] Yousefi S., Perozzo R., Schmid I., Ziemiecki A., Schaffner T., Scapozza L., Brunner T., Simon H.U. (2006). Calpain-mediated cleavage of Atg5 switches autophagy to apoptosis. Nat. Cell Biol..

[39] B’Chir W., Maurin A.C., Carraro V., Averous J., Jousse C., Muranishi Y., Parry L., Stepien G., Fafournoux P., Bruhat A. (2013). The eiF2alpha/ATF4 pathway is essential for stress-induced autophagy gene expression. Nucleic Acids Res..

